# Negative Regulator Nlrc3-like Maintain the Balanced Innate Immune Response During Mycobacterial Infection in Zebrafish

**DOI:** 10.3389/fimmu.2022.893611

**Published:** 2022-05-25

**Authors:** Liangfei Niu, Geyang Luo, Rui Liang, Chenli Qiu, Jianwei Yang, Lingling Xie, Kaile Zhang, Yu Tian, Decheng Wang, Shu Song, Howard E. Takiff, Ka-Wing Wong, Xiaoyong Fan, Qian Gao, Bo Yan

**Affiliations:** ^1^ Shanghai Public Health Clinical Center, Fudan University, Shanghai, China; ^2^ Shanghai Public Health Clinical Center, Key Laboratory of Medical Molecular Virology [Ministry of Education (MOE)/National Health Commission (NHC)/Chinese Academy of Medical Sciences (CAMS)], School of Basic Medical Sciences, Shanghai Medical College, Fudan University, Shanghai, China; ^3^ School of Medicine, Xizang Minzu University, Xianyang, China; ^4^ School of Life Sciences, Bengbu Medical College, Bengbu, China; ^5^ Medical College, China Three Gorges University, Yichang, China; ^6^ Department of Tuberculosis Control and Prevention, Shenzhen Nanshan Centre for Chronic Disease Control, Shenzhen, China; ^7^ Laboratorio de Genética Molecular, CMBC, Instituto Venezolano de Investigaciones Cientificas, Caracas, Venezuela

**Keywords:** mycobacterial infection, NLR, innate immunity, macrophage, neutrophil, inflammasome

## Abstract

The NOD-like receptors (NLRs) have been shown to be involved in infection and autoinflammatory disease. Previously, we identified a zebrafish NLR, *nlrc3-like*, required for macrophage homeostasis in the brain under physiological conditions. Here, we found that a deficiency of *nlrc3-like* leads to decreased bacterial burden at a very early stage of *Mycobacterium marinum* infection, along with increased production of pro-inflammatory cytokines, such as *il-1β* and *tnf-α*. Interestingly, myeloid-lineage specific overexpression of *nlrc3-like* achieved the opposite effects, suggesting that the impact of *nlrc3-like* on the host anti-mycobacterial response is mainly due to its expression in the innate immune system. Fluorescence-activated cell sorting (FACS) and subsequent gene expression analysis demonstrated that inflammasome activation-related genes were upregulated in the infected macrophages of *nlrc3-like* deficient embryos. By disrupting *asc*, encoding apoptosis-associated speck-like protein containing a CARD, a key component for inflammasome activation, the bacterial burden increased in *asc* and *nlrc3-like* double deficient embryos compared with *nlrc3-like* single deficient embryos, implying the involvement of inflammasome activation in infection control. We also found extensive neutrophil infiltration in the *nlrc3-like* deficient larvae during infection, which was associated with comparable bacterial burden but increased tissue damage and death at a later stage that could be alleviated by administration of dexamethasone. Our findings uncovered an important role of *nlrc3-like* in the negative regulation of macrophage inflammasome activation and neutrophil infiltration during mycobacterial infection. This highlights the importance of a balanced innate immune response during mycobacterial infection and provides a potential molecular basis to explain how anti-inflammatory drugs can improve treatment outcomes in TB patients whose infection is accompanied by a hyperinflammatory response.

## Introduction

Tuberculosis (TB) remains a major threat for public health, with about 9.9 million new TB patients and 1.3 million deaths per year, according to the 2021 WHO annual TB report (1). Patients who are immunodeficient or undergoing long term immunosuppressive therapy have an increased risk of *Mycobacterium tuberculosis* (*M.tb*) infections (2, 3), but a hyperinflammatory response to infection can induce extensive tissue damage (4–7). Thus, a balanced immune response is critical for successful host control of *M.tb* infections.

Innate immune cells are the first line of defense against *M.tb* infection, and macrophages are considered to be the first and the primary cell-type infected. Macrophages not only provide shelter for the immune escape of *M.tb*, but also mediate immune killing (8). ASC mediated macrophage IL-1β maturation has been shown to be a vital mechanism for macrophage restriction of *M.tb* (9, 10), and macrophages also mediate bactericidal activity *via* TNF-α and ROS signaling (11–16). However, excessive production of pro-inflammatory cytokines such as TNF-α will cause accelerated mycobacterial extracellular growth and subsequent tissue damage (7, 17). Therefore, the proper regulation of macrophage functions is critical for *M.tb* control.

Neutrophils are the most abundant infected innate immune cell type in the airways of pulmonary TB patients (18) but whether they are beneficial or detrimental during *M.tb* infection remains controversial. The impact of neutrophils on mycobacterial infection seems to depend upon the specific experimental conditions in the immune system microenvironment, the stage of the infection and the animal model used for the study (19, 20). It is generally thought that the involvement of neutrophils at a very early stage of infection has a positive effect that contributes to host control of a mycobacterial infection (19–22). However, neutrophils may also play a detrimental role during *M.tb* infection, serving as a “Trojan horse” for bacterial dissemination and also causing tissue damage as the disease progresses (6, 23–25). Similar to macrophages, neutrophils are versatile innate immune cells that can help or hinder host control of *M.tb* infection and therefore require balanced regulation.

NLRs are a class of intracellular receptors that typically include a C terminal leucine-rich repeats (LRRs) receptor domain, a central nucleotide-binding domain (NBD) and an N terminal effector domain (26, 27). Several NLRs, such as CIITA, NOD2, NLRC3 and NLRP10, are important sensors or regulators of the host anti-*M.tb* immune response (28–31). Most NLRs, especially inflammasome NLRs, are positive regulators of the immune response (26, 27). Upon binding to their ligands, NLRs can form oligomers *via* central NBD self-oligomerization and then recruit downstream effector proteins ASC and caspase-1 to form inflammasomes. The activated caspase-1 then cleaves pro-IL-1β to make mature IL-1β and promotes its subsequent secretion (26).

In contrast, some mammalian NLRs, such as NLRP10 and NLRC3, function as negative regulators rather than sensors of distinct stimuli (30–37). The zebrafish *nlrc3-like* is also a negative regulator of the Asc mediated inflammasome pathway and required for proper microglial colonization and maintenance (38, 39). Although the negative regulator NLRC3 has been shown to be involved in the adaptive immune response against *M.tb* infection (30), the impact of negative regulators such as NLRC3 on the innate immune response during mycobacterial infection has not been explored.

Zebrafish are an emerging model for host-mycobacterial interactions, especially for studying the intact innate immune system present in their embryonic stage, before their adaptive immunity has developed (40, 41). Using this model, we investigated the impact of the negative regulator *nlrc3-like* on the innate immune response to mycobacterial infection. The bacterial burden decreased significantly during the very early stages of *M. marinum* infection in *nlrc3-like* deficient embryos, accompanied by an increased production of cytokines with bactericidal effects. Using tissue specific overexpression of *nlrc3-like*, disruption of Asc signaling and FACS analysis, we demonstrate that the effects of *nlrc3-like* deficiency on the anti-mycobacterium response are principally mediated through the activation of the inflammasome in infected macrophages. In the *nlrc3-like* deficient larvae there was extensive neutrophilic infiltration, accelerated late-stage tissue damage and increased death rates, but this phenotype could be alleviated by the anti-inflammatory steroid dexamethasone (DEX).

Collectively, our findings uncover an important role for *nlrc3-like* in the negative regulation of both neutrophil infiltration and macrophage inflammasome activation during mycobacterial infection. This study demonstrates that the successful control of mycobacterial infection requires a balanced immune response. The results also suggest a molecular explanation for the benefit of anti-inflammatory drugs in patients with forms of tuberculosis associated with a hyperinflammatory state, such as tuberculosis meningitis or the immune reconstitution inflammatory syndrome (IRIS) in HIV/tuberculosis co-infections (42, 43).

## Methods

### Ethics Statement

All zebrafish were raised under standard conditions in compliance with laboratory animal guidelines for the ethical review of animal welfare (GB/T 35823-2018). All zebrafish experiments were approved by the Animal Care and Use Committee of the Shanghai Public Health and Clinical Center, Fudan University (2019-A016-01 & 2021-A043-01).

### Zebrafish Strains

Wild Type AB, *nlrc3-like^hkz6^* (39)*, asc^Δ31^ (asc^hkz7^)* (39)*, Tg(mfap4:GFP)^hkz020t^* (44) and *Tg(lyz:GFP)^nz117^* (45) were generated and maintained as previously described. All offspring from *nlrc3-like^hkz6^* were maintained at 32°C.

### *Nlrc3-like* Mutant Genotyping

Because only *nlrc3-like -/-* mutants show a phenotype (39), we could separate *nlrc3-like -/-* embryos from their siblings (*nlrc3-like +/+* and *nlrc3-like +/-*). *Nlrc3-like -/-* embryos were identified by differential interference contrast (DIC) imaging of apoptotic-body accumulation in the midbrain region at 3 days post fertilization (dpf) as previously described (39).

### Bacteria Strains and Zebrafish Infections

Wasabi and Tdtomato fluorescently labeled M strain *M. marinum* (ATCC BAA-535) were generated by Dr. Ramakrishnan’s group (40). Zebrafish embryo infections were performed by microinjection of *M. marinum via* the duct of Cuvier unless otherwise indicated (46). For live imaging and Sudan Black (SB) staining, infections were by subcutaneous injection (47). The infecting dose was 250 CFU, if not otherwise specified, but 400 CFU were used for experiments designed for analysis by FACS.

### Cell Culture and Infections

Raw264.7 (ATCC TIB-71) cells were cultured in DMEM (Biological Industries) containing 10% fetal bovine serum at 37°C and 5% CO2 with a density of 2 x 10^6^ cells per well in 6 well plates. For infections, the plated cells were infected with single-cell suspensions of H37Rv on the day after seeding, at a multiplicity of infection (MOI) of 10:1. After allowing 4 hours for phagocytosis, infected Raw264.7 cells were washed 3 times with 1 x PBS to remove extracellular bacteria and then replenished with fresh DMEM containing 10% FBS.

### Plasmid Construction and Expression

The full-length coding sequence of nlrc3*-like* was obtained from the *pTol2-coronin1a-nlrc3-like* plasmid and subcloned into the *pTol2-β-actin* vector to generate *pTol2-β-actin-nlrc3-like* (39). The control plasmids *pTol2-β-actin-6xmyc* and *pTol2-coronin1a-6xmyc* were generated similarly (39). Plasmid expression in zebrafish embryos was achieved by microinjection of 2 nL per embryo of each plasmid (10 ng/μL) together with transposase mRNA (100 ng/μL) at the single-cell stage.

### Quantification of Bacterial Burden

Fluorescently labeled bacteria were quantified by measuring the integrated fluorescent intensity with Fiji ImageJ software.

### Statistical Analysis of Granuloma-Like Structure Size

Images were processed by Fiji ImageJ software using the “Analyze Particles” function.

### Time-Lapse Imaging

Time-Lapse imaging for the neutrophil recruitment to the infection site was conducted and processed as previous described (48).

### Fluorescence-Activated Cell Sorting (FACS)

FACS was performed as previously described (49). For each experimental condition, 25 embryos were collected in a 1.5 mL microtube with 0.5 mL PBS. Embryos were homogenized by 5 passages through a 22-guage needle attached to a 5 ml syringe. The resultant homogenate was centrifuged at 300g for 5 minutes at 4°C, and the pellet was dissociated with 2 mL pre-warmed TrypLE (ThermoFisher Scientific) for 20 minutes at 32°C. Cells were washed by adding 2 mL pre-cooled 2% BSA/PBS solution and collected with a 100-μm strainer (Miltenyi). The passed-through cells were collected by centrifugation at 400 g for 5 minutes at 4°C and resuspended in 200 μL 1% BSA/PBS. The samples were pre-cooled on the collecting platform of a Bectin Dickinson FCSAria instrument before sorting. Sorted samples were collected into 4 μL lysis buffer of the QIAseq FX Single Cell RNA Library Kit (QIAGEN) in a 96-well plate.

### RNA Extraction and Quantitative RT-PCR

Whole zebrafish embryos were homogenized by five passages through a 27-G needle in 350 μL lysis buffer, and RNA was extracted using the RNeasy Mini Kit (QIAGEN). cDNA was synthesized from 0.5 ~ 1 μg RNA using the PrimeScript RT reagent Kit (Takara). For sorted cells, RNA extraction and cDNA preparation were performed with the QIAseq FX Single Cell RNA Library Kit (QIAGEN), according to the manufacturer’s instructions. For infected Raw264.7 cells, samples were collected at 4, 10, 24, 72 hours post infection (hpi). Cells were washed with 1x PBS and lysed in 1mL TRizol (ThermoFisher Scientific) for future RNA isolation. Quantitative RT-PCR was performed with SYBR Green Supermix (Promega) on a CFX96 Real-Time System instrument (Bio-Rad). The primers used for quantitative RT-PCR are listed in **Supplementary Table 1**.

### Whole Mount *In Situ* Hybridization (WISH)

*Il-1β* WISH was performed as previous described (48).

### Double Staining for RNA (*il-1β*) and Protein (GFP)

Two color RNA and protein staining was performed as previously reported (39). WISH staining was first developed with a TSA plus cyanine 3 kit (Akoya, NEL744001KT). Anti-GFP staining was performed with goat polyclonal anti-GFP primary antibodies (Abcam, ab6658, 1:500) and AF488 donkey anti-goat (Invitrogen, A11055, 1:500) secondary antibodies.

### Sudan Black (SB) Staining

SB staining was performed as previously described (39).

### DEX Treatment

100 μM DEX treatment was started at 1 day post infection (dpi) and continued until all larvae had died. The larvae were incubated in egg water containing 100 μM DEX for 12 hours and changed to drug free egg water for 12 hours daily.

### Survival Curve

The statistical analysis and infection methods were previously described in detail (50). Briefly, embryos were infected with *M. marinum*: *wasabi* (~ 250 CFU), and the daily number of embryos deaths was recorded.

### Histology of Zebrafish Embryos

Zebrafish embryos at 6 dpi were fixed for at least 24 hours in 4% paraformaldehyde. Paraffin embedding, serial sectioning (4~5 μm), and subsequent H&E staining or acid-fast staining were performed as previously reported (50).

### Statistical Analysis

Statistical analyses were performed using GraphPad Prism software. All data are shown as the mean ± SEM. Differences were analyzed with unpaired two-tailed t test for comparisons between two groups, and One-Way ANOVA for multiple groups. Statistical significance was indicated as follows: *p ≤ 0.05; **p ≤ 0.01; ***p ≤ 0.001; ns, not statistically significant (p>0.05).

## Results

### The Defect of *nlrc3-like* Facilitate the Early Control of Mycobacterial Infection

**Figure 1:**
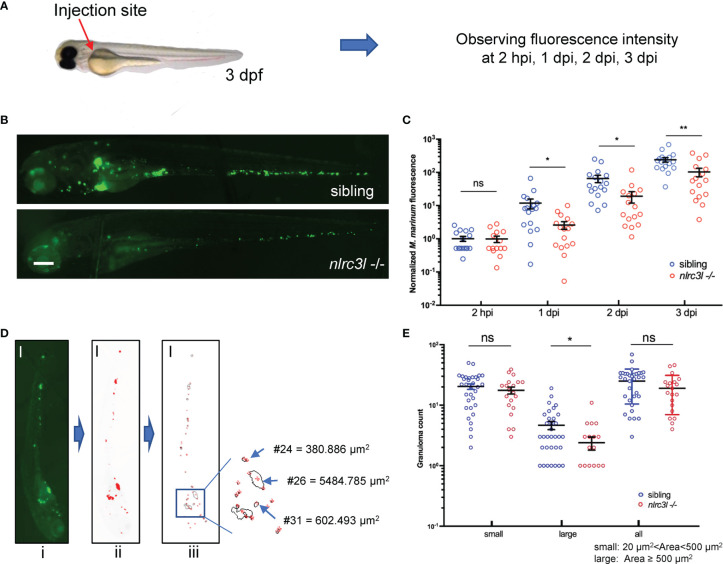
*nlrc3-like* deficiency promotes host control of the mycobacterial proliferation during early infection. **(A)** Zebrafish embryo infections and bacterial fluorescence intensity. The red arrow indicates the duct of Cuvier, the site where bacteria were injected. **(B)** Representative images for *M. marinum*:*Wasabi* infected embryos at 3 dpi. **(C)** Statistics of relative green fluorescence intensity at the indicated time points taken from two independent biological replicates. **(D)** Process for calculating granuloma-like structure in zebrafish embryos: i original image; ii. the image after fluorescence-pixelation processing – the area of ​​the red pixels represents the relative size of each granuloma; iii. Calculation of the area of ​​the red pixels. **(E)** Graph showing the number of large, small and all granuloma-like structures in *nlrc3-like -/-* and control embryos. **(B, D)** Scale bar = 200 μm *p ≤ 0.05; **p ≤ 0.01; ns, not statistically significant (p>0.05)..

It has been shown that NLRC3, a negative regulator NLR, is involved in the adaptive immune response against *M.tb* infection (30), but its effect on the innate immune regulation during mycobacterial infection has not been explored. To investigate whether zebrafish negative regulatory *nlrc3-like* is also involved in the anti-mycobacterium innate immunity, we studied a *nlrc3-like* mutant (40, 41) in the embryo stage, when innate immunity is intact but adaptive immunity has not yet developed. Zebrafish embryos were infected with fluorescence-labeled *M. marinum*, and the relative fluorescence intensity was monitored to quantify the bacterial load of each embryo (**Figure 1A**). Compared with the sibling controls (*nlrc3-like +/+* and *nlrc3-like +/-*), the bacterial burden in *nlrc3-like -/-* embryos was significantly decreased at 1 dpi, 2 dpi and 3 dpi (**Figures 1B, C**). We also found that there were fewer large granuloma-like structures in the *nlrc3-like* mutants (**Figures 1D, E**).

### *nlrc3-like* Deficiency Boosts the Expression of Mycobacterial Elicited Pro-Inflammatory Genes

**Figure 2:**
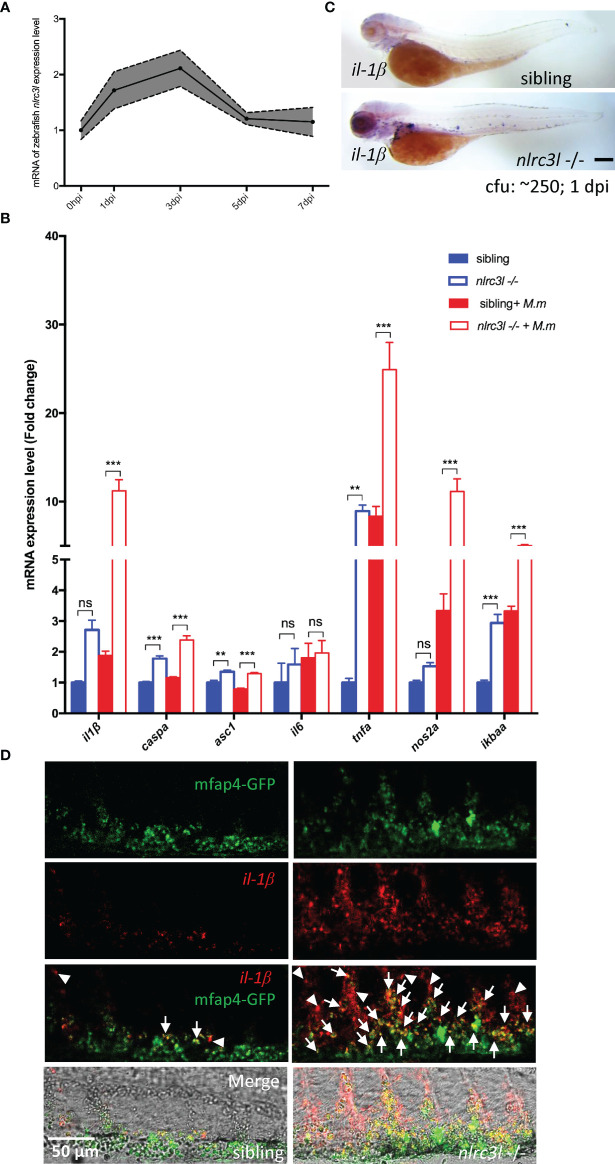
*nlrc3-like* deficiency upregulates the expression of inflammatory genes during mycobacterial infection. **(A)** Quantitative RT-PCR showing expression of *nlrc3-like* in 3 dpf zebrafish embryos at 0, 1, 3, 5 and 7 days post infection with *M. marinum*:*Wasabi.* **(B)** Quantitative RT-PCR showing expression of multiple inflammatory genes at 1 dpi. The values shown represent an average of three independent biological experiments, 25 embryos per group in each experiment. **(C)** *il-1β* WISH shows significantly increased *il-1β* expression in *nlrc3-like* deficient mutants compared with their siblings at 1 dpi. Scale bar = 200 μm. **(D)** Co-staining of *il-1β* WISH (red) and anti-GFP antibody (green). White arrows point to co-staining signals of mfap4-GFP and *il-1β*, and white arrow heads point to the *il-1β* only signals. Scale bar = 50 μm **p ≤ 0.01; ***p ≤ 0.001; ns, not statistically significant (p>0.05).

Pro-inflammatory factors are required for the host anti-mycobacterial response and we wondered whether they are upregulated in the *nlrc3-like* deficient mutant. *nlrc3-like* mRNA expression increased during the first 3 dpi and then gradually returned to the basal level at 5 dpi (**Figure 2A**). We found that *il-1β, tnf-α* and other pro-inflammatory genes were up-regulated in the *nlrc3-like -/-* mutant (**Figure 2B**). We then confirmed the upregulation of *il-1β* mRNA by whole mount *in situ* hybridization (WISH) in the *nlrc3-like* deficient embryos at 1 dpi (**Figure 2C**) and also found that a large portion of *il-1β* mRNA co-localized with mfap4-GFP staining (**Figure 2D**), suggesting that macrophages are an important source for *il-1β* during mycobacterial infection.

### The Impact of *nlrc3-like* on Early Control of Mycobacterial Proliferation Is Mediated Principally by Innate Immune Cells

**Figure 3:**
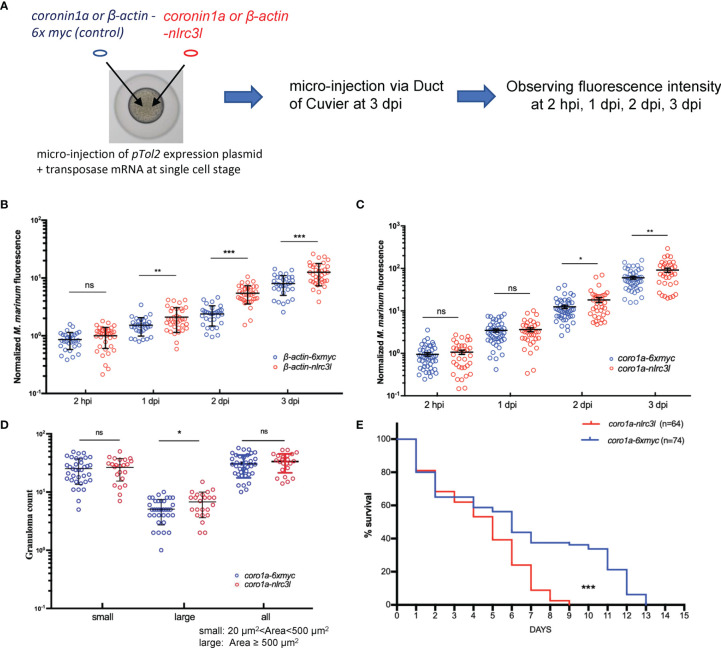
The impact of *nlrc3-like* on host defense against mycobacterial infection was mainly mediated by innate immune cells. **(A)** Schematic view of the construction of *nlrc3-like* overexpressing embryos and the *M. marinum* infection assay. *pTol2* plasmids were injected at the single-cell stage and subsequently the grown embryos were infected *via* the duct of Cuvier at 3 dpf. Injections of *pTol2-β-actin-nlrc3-like* (*β-actin-nlrc3l*) and *pTol2-coronin1a-nlrc3-like* (*coro1a-nlrc3l*) generated embryos overexpressing *nlrc3-like* in all cells or only in myeloid cells, respectively. Black arrows indicate injection sites. **(B)** Graph of the relative green fluorescence intensity of the *β-actin-nlrc3l* group and the *β-actin-6xmyc* group at the indicated time points. **(C)** Graph of the relative green fluorescence intensity of the *coro1a-nlrc3l* and *coro1a-6xmyc* embryos at the indicated time points. **(D)** Graph showing the number of granuloma-like structures in the *coro1a-nlrc3l* and the *coro1a-6xmyc* embryos. **(E)** Survival curve of *coro1a-nlrc3l* and *coro1a-6xmyc* embryos. The data shown represents the averages from two independent biological replicates *p ≤ 0.05; **p ≤ 0.01; ***p ≤ 0.001; ns, not statistically significant (p>0.05).

While *nlrc3-like -/-* embryos had a reduced bacterial burden at early time points post infection, overexpression of *nlrc3-like* produced a significantly increased bacterial burden, as measured by bacterial fluorescence intensity (**Figures 3A, B**; **Supplementary Figure 1A**). To investigate whether the action of *nlrc3-like* during mycobacterial infection was mediated by innate immune cells or non-immune cells, we generated zebrafish that overexpressed *nlrc3-like* only in myeloid cells. The increased bacterial fluorescence showed that the myeloid cell specific overexpression of *nlrc3-like* resulted in an elevated bacteria burden, an increased number of large granuloma-like structures and reduced survival, similar to the effects seen in embryos with whole-body overexpression of *nlrc3-like* (**Figures 3C-E**; **Supplementary 1B**). These results suggest that the impact of *nlrc3-like* on mycobacterial proliferation *in vivo* is principally mediated *via* its expression in innate immune cells.

### *nlrc3-like* Deficiency Promotes Asc Mediated Inflammasome Activation

**Figure 4:**
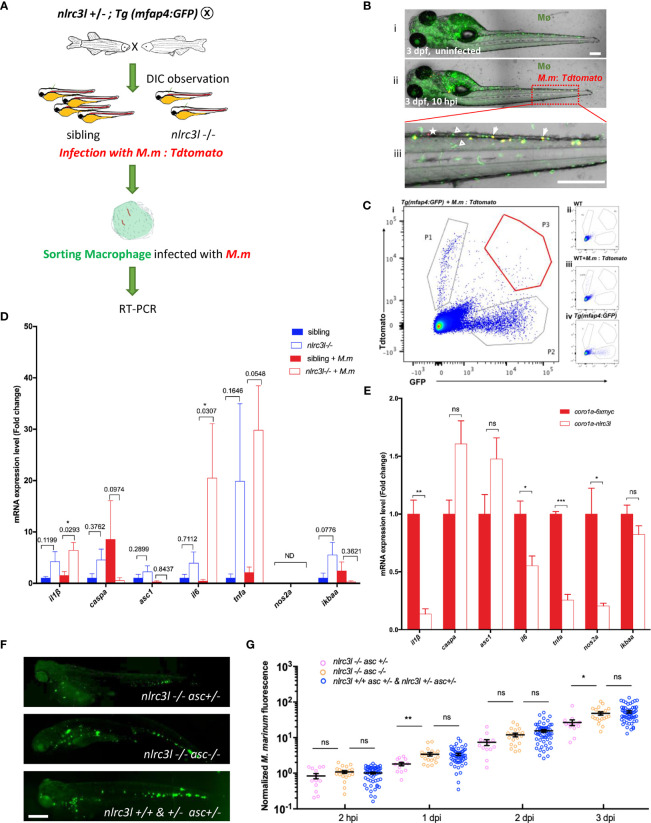
*nlrc3-like* deficiency promotes the activation of macrophage inflammasome. **(A)** Schematic view of FACS of mfap4-GFP^+^ macrophages with phagocytosed Tdtomato^+^ *M. marinum* and subsequent detection of the expression of inflammation-related genes by RT-PCR. **(B)** A representative image of zebrafish embryos infected with *M. marinum:Tdtomato* at 10 hpi. i uninfected *Tg (mfap4-GFP)* embryo; ii. *Tg (mfap4-GFP)* embryo infected with *M. marinum:Tdtomato*; iii. Enlarged view of caudal hematopoietic tissue (CHT) region in ii. white arrows indicate macrophages with phagocytosed bacteria; white triangles indicate macrophages without phagocytosed bacteria; white asterisks, free *M. marinum*. **(C)** Gating strategy for FACS. i: Sample: *Tg(mfap4: GFP)* embryos infected with *M. marinum:Tdtomato*; ii. uninfected wild-type zebrafish; iii. Tdtomato single color control, wild-type zebrafish infected with M. marinum:Tdtomato; iv. GFP single color control, uninfected *Tg(mfap4: GFP)*. P1, free *M. marinum:Tdtomato*; P2, mfap4-GFP+ macrophages; P3, mfap4-GFP+ macrophages with phagocytosed *M. marinum:Tdtomato*. **(D)** Expression of pro-inflammatory genes in macrophages at 10 hpi. The values represent the average of three independent biological repeats, 25 embryos per group in each repeat. **(E)** The expression of pro-inflammatory genes in embryos with overexpressing *nlrc3-like* in myeloid cells. **(F)** Representative images of *M. marinum:wasabi* proliferation in *nlrc3-like* -/-, *asc -/- nlrc3-like -/-*, and their nonmutant siblings. **(G)** Graph of green fluorescence intensity in *nlrc3-like -/-*, *asc -/- nlrc3-like -/-*, and their nonmutant siblings at the indicated time points. The values shown are the combined results of two independent experimental replicates. **(B, F)** scale bar = 200 μm *p ≤ 0.05; **p ≤ 0.01; ***p ≤ 0.001; ns, not statistically significant (p>0.05).

Next, we explored how *nlrc3-like* acts on innate immune cells during mycobacterial infection. Because macrophages are the primary immune cell phagocytizing mycobacteria *in vivo*, we used FACS to collect macrophages containing phagocytosed fluorescent mycobacteria for gene expression analysis (**Figure 4A**). Through continuous observation we found that the number of infected macrophages peaked at approximately 10 hpi and therefore chose this time point for FACS analysis of infected macrophages (**Figures 4B, C**). Similar to the results with whole embryos, at 10 hpi the macrophages of *nlrc3-like -/-* mutants showed significantly increased expression of several inflammasome priming related genes such as *il-1β* and *il-6* (**Figure 4D**). We then evaluated the expression of these pro-inflammatory factors in embryos with myeloid specific overexpression of *nlrc3-like*, and as expected, found dramatically decreased expression of pro-inflammatory genes (**Figure 4E**).

Full activation of the inflammasome in macrophages requires both priming (signal 1) and activation of the inflammasome complex assembly (signal 2) (51). Therefore, if *nlrc3-like* deficiency causes the inflammasome activation, there should be an increased in mature Il-1β protein. However, due to the limited antibody resources in zebrafish, it was not possible to detect and measure the cleaved form of Il-1β. Instead, we disrupted the inflammasome activation required adaptor gene *asc* and found that the decreased bacterial burden seen in the *nlrc3-like* deficiency embryos was reversed in *nlrc3-like* and *asc* double mutants (**Figures 4F, G**). This suggested that the absence of *nlrc3-like* promotes the early control of mycobacterial proliferation *via* the activation of the *asc* mediated macrophage inflammasome pathway.

### *nlrc3-like* Deficiency Accelerates Neutrophil Recruitment

**Figure 5:**
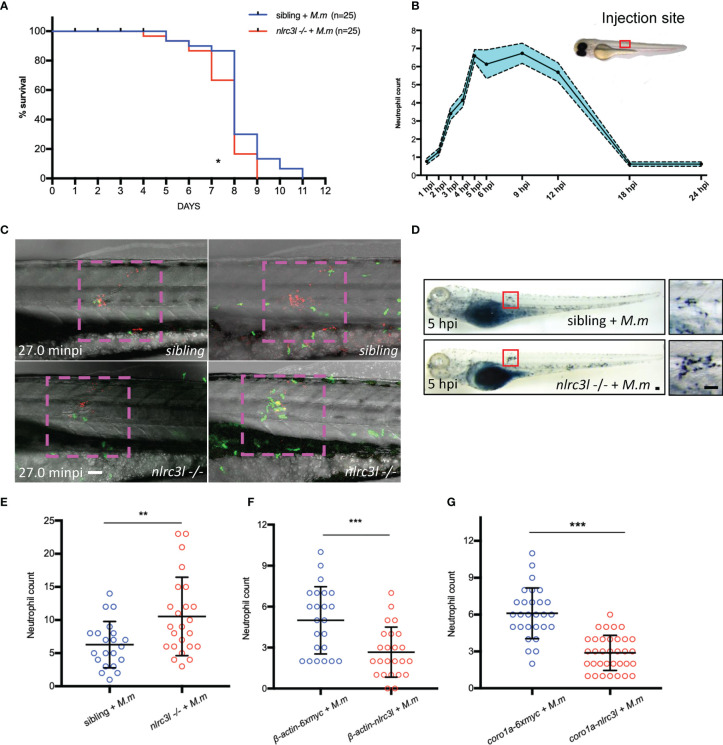
*nlrc3-like* deficiency promotes neutrophil recruitment. **(A)** Survival curves of *nlrc3-like -/-* and non-mutant embryos after *M. marinum* infection, based on results from two independent biological replicates. **(B)** Numbers of neutrophils recruited to infection sites at the indicated time points. **(C)** Live imaging of neutrophils after *M. marinum* infection. 3 dpf *Tg (lyz-GFP)* zebrafish embryos were infected with *M. marinum:Tdtomato*. Time interval, 1 min 30 s. The data is representative of three independent biological replicates. **(D)** SB staining showing neutrophil recruitment to infection sites. The left panel shows the whole embryo while the right panel shows magnification of the area in the red box used for counting neutrophils. **(E)** Graph of neutrophils counted in **(D, F)** Graph of the neutrophils counted after SB staining of *β-actin-nlrc3l* (whole body expression of *nlrc3-like*) and *β-actin-6Xmyc* embryos. **(G)** Graph of neutrophils counted after SB staining in *coro1a-nlrc3l* (myeloid expression of *nlrc3-like*) and *coro1a-6Xmyc* embryos **(C, D)**. Scale bar = 50 μm *p ≤ 0.05; **p ≤ 0.01; ***p ≤ 0.001.

Unexpectedly, despite the lower bacterial burden in *nlrc3-like -/-* embryos during early infection, their survival curve was slightly but significantly worse than that of the nonmutant embryos after infection, of which very few died in the uninfected group (**Figure 5A** and data not shown). Neutrophils that are recruited soon after infection have bactericidal activity (6, 19, 21). Recruitment of neutrophils to the site of infection peaked at 5 hpi (**Figure 5B**), and live imaging *in vivo* revealed a more intense neutrophil infiltration in *nlrc3-like -/-* embryos (**Figure 5C**; **Supplementary Video 1**), as confirmed by SB staining (**Figures 5D, E**). In order to clarify whether the increased neutrophilic infiltration was mediated by immune or non-immune cells, we compared the recruitment of neutrophils in embryos with whole body overexpression of *nlrc3-like* to embryos in which only the myeloid cells overexpressed *nlrc3-like*. We found that the neutrophil recruitment was significantly decreased in both groups of embryos (**Figures 5F, G**), suggesting that, as with the increased bacterial burden, this effect of overexpressed *nlrc3-like* is mediated principally through its expression in myeloid cells. Taken together, these results suggest that the early restriction of mycobacterial proliferation in the setting of *nlrc3-like* deficiency may be partly due to the increased neutrophil recruitment to the site of infection.

### Loss of *nlrc3-like* Results in Excessive Immune Cell Infiltration and Tissue Damage at the Later Stages of Mycobacterial Infection

**Figure 6:**
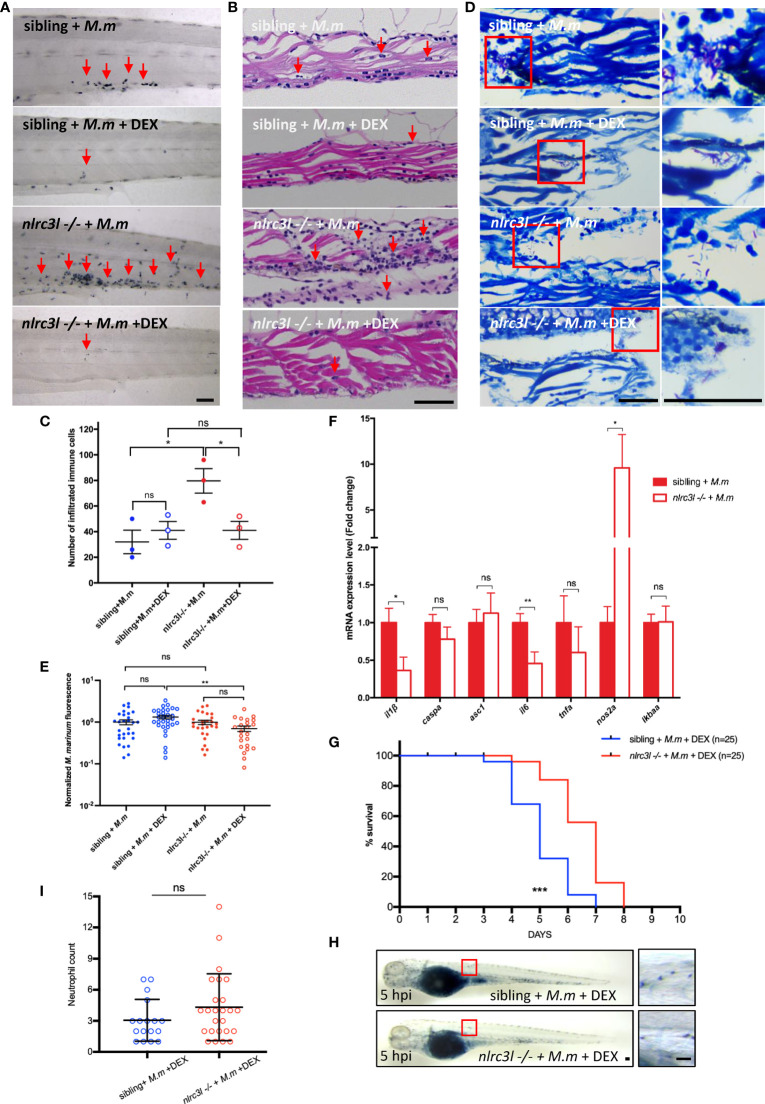
*nlrc3-like* deficiency leads to excessive neutrophils infiltration and tissue damage at later stages of infection. **(A)** SB staining of the tail region in different groups of zebrafish embryos. **(B)** H&E staining of paraffin sections of the caudal vein plexus. **(C)** Quantification of the number of infiltrated immune cells in **(B, D)** Acid fast staining of paraffin sections in the caudal vein plexus. **(E)** Statistics of normalized bacterial fluorescence intensity at late stage of infection. **(F)** Quantitative RT-PCR assessed the expression of multiple cytokines at a late stage of infection (6 dpi). The values are averages from three independent biological repeats, 25 embryos per group in each repeat. **(G)** Survival curves of infected zebrafish with DEX treatment. The data is from one of two similar independent biological replicates **(H)**. SB staining showing neutrophil recruitment to infection sites at 5 hpi. The left panel shows the whole embryo while the right panel shows an enlargement of the region in the red box that was used for counting neutrophils. **(I)** Graph showing the number of neutrophils, counted as in **(H)**. The data shown was combined from three independent biological repeats. **(A, B, D, H)** Scale bar = 50 μm *p ≤ 0.05; **p ≤ 0.01; ***p ≤ 0.001; ns, not statistically significant (p>0.05).

We then examined the effects of *nlrc3-like* deficiency on the later stages of infection. Compared to the sibling control, there was a significantly increased immune cell infiltration, especially of neutrophils, in the caudal vein plexus in *nlrc3-like -/-* embryos (**Figures 6A-C**). However, the bacterial burdens were comparable between *nlrc3-like -/-* embryos and their siblings, as measured by acid-fast staining and bacterial fluorescence (**Figures 6D, E**). The expression of the pro-inflammatory *nos2a* mRNA was greatly increased (**Figure 6F**). Interestingly, the increased NO signaling was coupled with decreased expression of pro-inflammatory cytokines *il-1β* and *il-6* (**Figure 6F**). A similar expression pattern of inflammatory genes was also observed in H37Rv infected Raw264.7 macrophages (**Supplementary Figure 2**).

### Treatment With Anti-Inflammatory Corticosteroids Relieves the Excessive Neutrophil Infiltration and Prolongs the Survival of *nlrc3-like* Deficient Mutant Embryos

To separate the beneficial, antibacterial effects of *nlrc3-like* deficiency from the destructive effects of hyperinflammation, we administered the anti-inflammatory corticosteroid DEX beginning at 1 dpi. The *nlrc3-like -/-* embryos treated with DEX survived significantly longer than their non-mutant siblings given DEX (**Figure 6G**). Although the neutrophilic infiltration and tissue damage were decreased in both groups, the reduction was greater in the *nlrc3-like -/-* mutants, thereby eliminating any statistical difference between the two groups (**Figures 6A, C**). A similar phenotype was also noted in the neutrophil recruitment assay using subcutaneous infection (**Figures 6H, I**). Together, these results suggest the detrimental, tissue damaging effects of *nlrc3-like* deficiency on the later stages of mycobacterial infection are most likely due to the excessive immune cell infiltration rather than increased bacterial burden.

## Discussion

A balanced immune response is critical for successful host control of mycobacterial infections. An insufficient immune response will be unable to limit the infection while an excessive immune response will cause tissue damage and bacterial dissemination (7, 52, 53). NLRs are a class of intracellular receptors, the majority of which function by detecting external stimuli and activating downstream inflammasome pathways (26). Several NLRs, however, appear to play a regulatory function in autoimmune and infectious diseases by mediating immune homeostasis (26). Compared with inflammasome NLRs, the role of regulatory NLRs in mycobacterial infections has not been well studied, and their possible involvement in adaptive immune regulation has been merely implied (30, 31). To learn about how regulatory NLRs affect innate immune cells involved in the host anti-mycobacterial response, we used *M. marinum* infections of zebrafish embryos as a model. Zebrafish embryos are useful because they have intact innate immunity but have not yet developed an adaptive immune system. By studying the embryos of zebrafish lacking *nlrc3-like*, a negative regulatory NLR, we could show that it is required for the regulation of innate immunity during mycobacterial infection. Negative regulatory NLRs could also play a key role in the innate immune response to tuberculosis infections in humans, although the specific details may be different.

**Figure 7:**
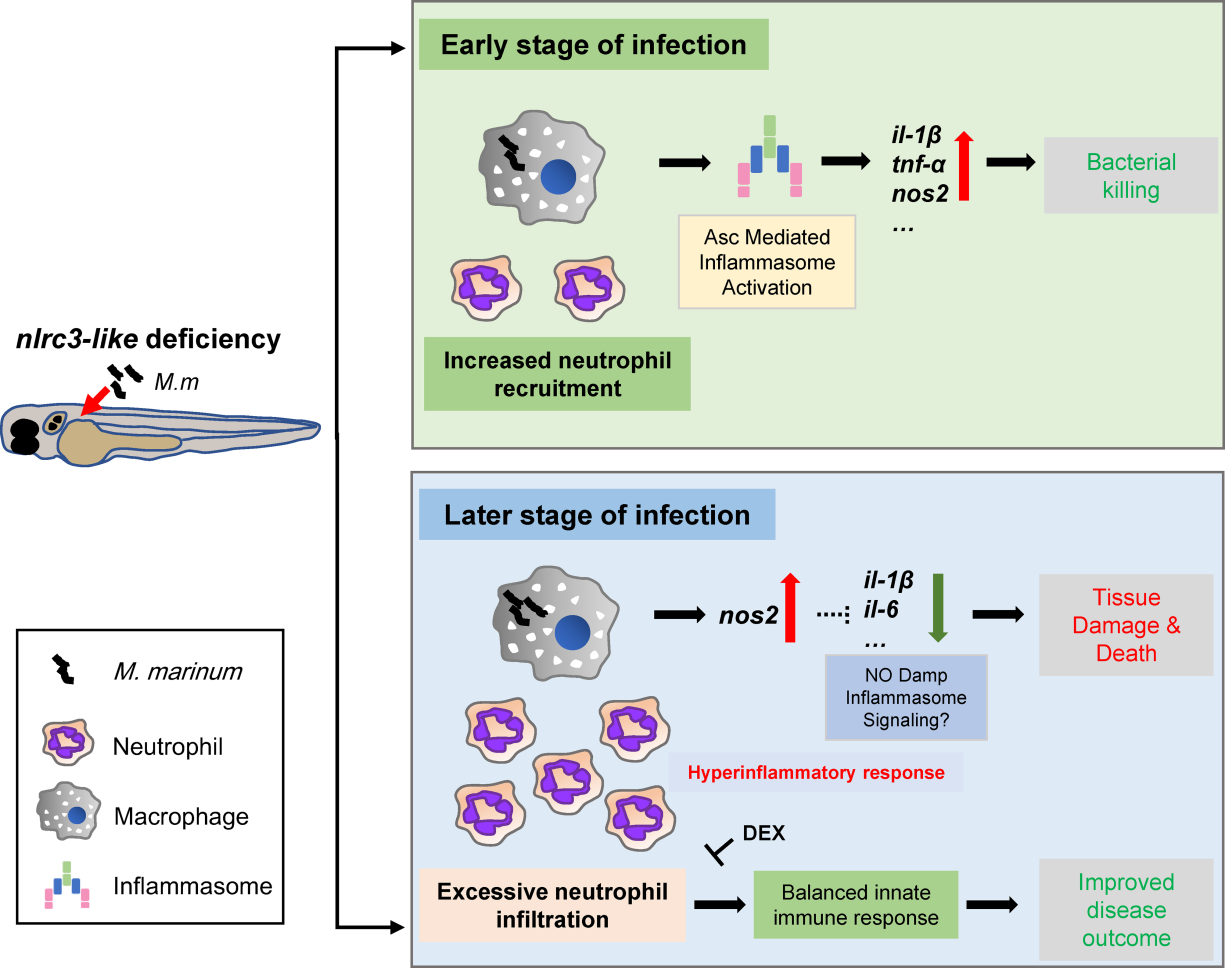
Model for the impact of negative regulator NLR *nlrc3-like* on innate immune responses during mycobacterial infection. During the early stage of infection, the defect of *nlrc3-like* can boost the expression of mycobacterial elicited pro-inflammatory genes *via* Asc mediated inflammasome activation in infected macrophages, which results in a bacterial killing effect accompanied by an increased neutrophil recruitment. However, during the late stage of infection the host shifts into a hyperinflammatory status with uncontrolled excessive neutrophil infiltration and increased tissue damage and death. The administration of DEX can block the excessive inflammatory response and improve disease outcome.

While cytokines with bactericidal effects are essential for host control of mycobacterial infections, and defects in cytokines such as IL-1β or TNF-α can lead to the death of infected individuals (9, 52, 53), excessive production of these same cytokines is detrimental to the host (7, 17, 52). In this study, the *nlrc3-like* defect was associated with increased expression of pro-inflammatory cytokines and reduced bacterial proliferation during the early stages of infection (**Figure 7**). At later stages of infection, however, the *nlrc3-like -/-* embryos showed uncontrolled immune cell infiltration and increased tissue damage (**Figure 7**).

The tissue damage may have been the result of the increased expression of *nos2a* mRNA (54). High levels of Nos2a could result in increased nitric oxide (NO) production, leading to enhanced bactericidal activity and increased tissue damage in *nlrc3-like -/-* mutant embryos (54–56). This would explain how a lower bacterial burden in *nlrc3-like -/-* embryos at early stage is associated with reduced long-term survival. Interestingly, the increased *nos2a* mRNA was coupled with a decreased expression of pro-inflammatory cytokines *il-1β* and *il-6* (**Figure 6D**), perhaps because increased NO in the mutant damped the activation of inflammasome as has been previously described (5, 56). Similar expression patterns of inflammatory genes were also observed in the H37Rv infected Raw264.7 macrophages (**Supplementary Figure 2**). Thus, it appears that the negative regulatory protein Nlrc3-like is indispensable for controlling the inflammatory response and thereby limiting tissue damage that can occur at late stages of mycobacterial infections.

Mycobacterial infections activate macrophage inflammasome components such as NLRP3 and AIM2 (10, 57), which require ASC as a key adaptor molecule for the successful initiation of the host anti-pathogen response. Without tight regulation however, this can cause excessive inflammation and collateral tissue damage (5). In this study, we demonstrated that *nlrc3-like* deficiency leads to uncontrolled Asc dependent inflammasome activation. Interestingly, we also noted that several inflammasome-independent genes were also elevated, suggesting the negative regulating role of *nlrc3-like* seem to be non-specific. In mammals, several NLRs, such as NLRP10 and NLRC3, function as negative regulators to attenuate inflammatory signaling (26, 30, 31, 34, 35), and NLRC3 has recently been reported to function as a negative regulator for CD4^+^ T cell activation during *M.tb* infection (30). In addition to its regulation of the adaptive immune response, NLRC3 has also been implicated in damping innate immune signaling after challenges with LPS and herpes simplex virus 1 (36, 37). While both the zebrafish Nlrc3-like and the mammalian NLRC3 contain a NACHT domain, the zebrafish protein lacks the leucine rich repeats. In spite of this difference, it would be interesting to see if mammalian NLRC3 functions in a similar manner to negatively regulate the innate immune response during *M.tb* infection.

Neutrophil infiltration during mycobacterial infection is a double edged sword (19). At early stages of infection we observed significantly increased neutrophil infiltration and significantly decreased bacterial burden in *nlrc3-like* deficient embryos. This might be the result of the increased *il-1β* production during the early infection, as *il-1β* has been shown to be a potent stimulus for neutrophil recruitment (48, 58). Another possibility is that the elevated *tnf-α* expression leads to macrophage lysis, and the intracellular material released by lytic cell death functions as a neutrophil attractant (39, 47). This is consistent with a recent study showing that macrophage pyroptosis is beneficial for host control of mycobacterial infection (59).

However, while the excessive immune cell infiltration seen in *nlrc3-like* deficient embryos may help control the infection at early stages, the bacterial burdens do not show significant difference at late stages. The excessive immune cell infiltration also leads to tissue damage at later stages that was ameliorated by the application of DEX. These data suggest the initial control of infection is accomplished by increased inflammation but too much inflammation eventually leads to a relatively attenuated control that allows the lower early bacterial burden to catch up at later stages. Although the relative lower survival of *nlrc3-like -/-* mutant embryos after *M. marinum* infection was reverted by the application of DEX, both treated groups had worse survival curves than untreated WT embryos. This suggests that DEX can ameliorate the hyperinflammation induced tissue damage during mycobacterial infection but also has detrimental side effects, although these that might be reduced by optimizing the dose and timing of the drug. One reason that the role of neutrophils in mycobacterial infections remains controversial is because contradictory data has been generated from studies in different models systems, such as human, mouse and zebrafish (19–21, 23). In our system, we observed both protective and detrimental effects, which suggest that the role of neutrophils during mycobacterial infection may depend upon the specific experimental conditions, such as the stage of infection and the host immune status.

Collectively, our results show that NLR Nlrc3-like regulates the host anti-mycobacterial response by limiting macrophage activation and neutrophil infiltration, thus highlighting that the successful control of mycobacterial infection requires a balanced immune response. Further experiments are needed to define all the components of the immune system that are regulated by Nlrc3-like during mycobacterial infection, but our findings suggest that the role of negative NLR regulators in innate immune regulation in human TB warrants investigation. The results also provide a molecular explanation for the beneficial effect of anti-inflammatory drugs in patients whose *M. tb* infection is associated with a hyperinflammatory state, such as seen in tuberculosis meningitis and the IRIS in HIV/tuberculosis co-infections.

## Data Availability Statement

The original contributions presented in the study are included in the article/**Supplementary Material**. Further inquiries can be directed to the corresponding authors.

## Ethics Statement

All zebrafish were raised under standard conditions in compliance with laboratory animal guidelines for the ethical review of animal welfare (GB/T 35823-2018). All zebrafish experiments were reviewed and approved by the Animal Care and Use Committee of the Shanghai Public Health Clinical Center, Fudan University (2019-A016-01 & 2021-A043-01).

## Author Contributions

Conceptualization: HT, QG, and BY. Data curation: LN, GL, and RL. Formal analysis: LN, GL, RL, CQ, DW, and SS. Funding acquisition: QG and BY. Investigation: LN, GL, RL, CQ, JY, LX, KZ, YT, and BY. Methodology: LN, GL, DW, SS, and BY. Project administration: QG and BY. Resources: K-WW, XF, QG, and BY. Supervision: QG and BY. Validation: LN and GL. Visualization: LN, GL, and BY. Writing - original draft: LN, GL, and BY. Writing - review and editing: all co-authors. All authors contributed to the article and approved the submitted version.

## Funding

This work was supported by the National Natural Science Foundation of China (81801977 to B.Y.; 81661128043, 81871625 to Q.G.), Shanghai Key Clinical Specialty Construction Project (Tuberculosis) (shslczdzk03002), the Technology Service Platform for Detecting High level Biological Safety Pathogenic Microorganism Supported by Shanghai Science and Technology Commission (21DZ2291300), Shanghai Public Health Clinical Center Intramural Research Funding (KY-GW-2022-01), the Outstanding Youth Training Program of Shanghai Municipal Health Commission (2018YQ54 to B.Y.), and the Shanghai Sailing Program (18YF1420400 to B.Y.).

## Conflict of Interest

The authors declare that the research was conducted in the absence of any commercial or financial relationships that could be construed as a potential conflict of interest.

## Publisher’s Note

All claims expressed in this article are solely those of the authors and do not necessarily represent those of their affiliated organizations, or those of the publisher, the editors and the reviewers. Any product that may be evaluated in this article, or claim that may be made by its manufacturer, is not guaranteed or endorsed by the publisher.
