# Supplementary material for: Negative Regulator Nlrc3-like Maintain the Balanced Innate Immune Response During Mycobacterial Infection in Zebrafish
Source: Front Immunol. 2022 May 25;13:893611. doi: 10.3389/fimmu.2022.893611 (PMC9174460; doi:10.3389/fimmu.2022.893611)
Supplement: Supplementary file 1 [file DataSheet_1.docx]

**Supplementary Data**

**B**

**A**

**
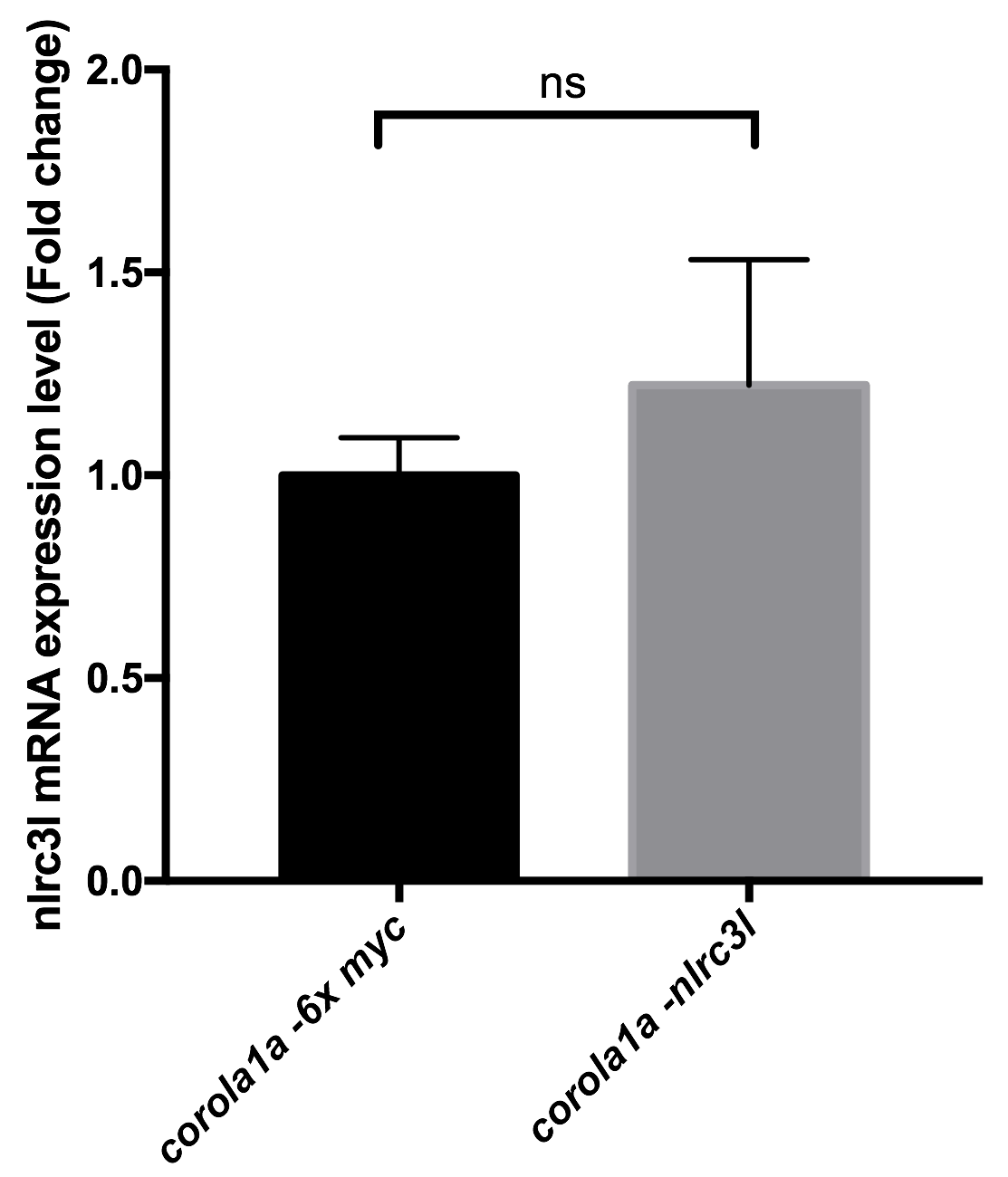

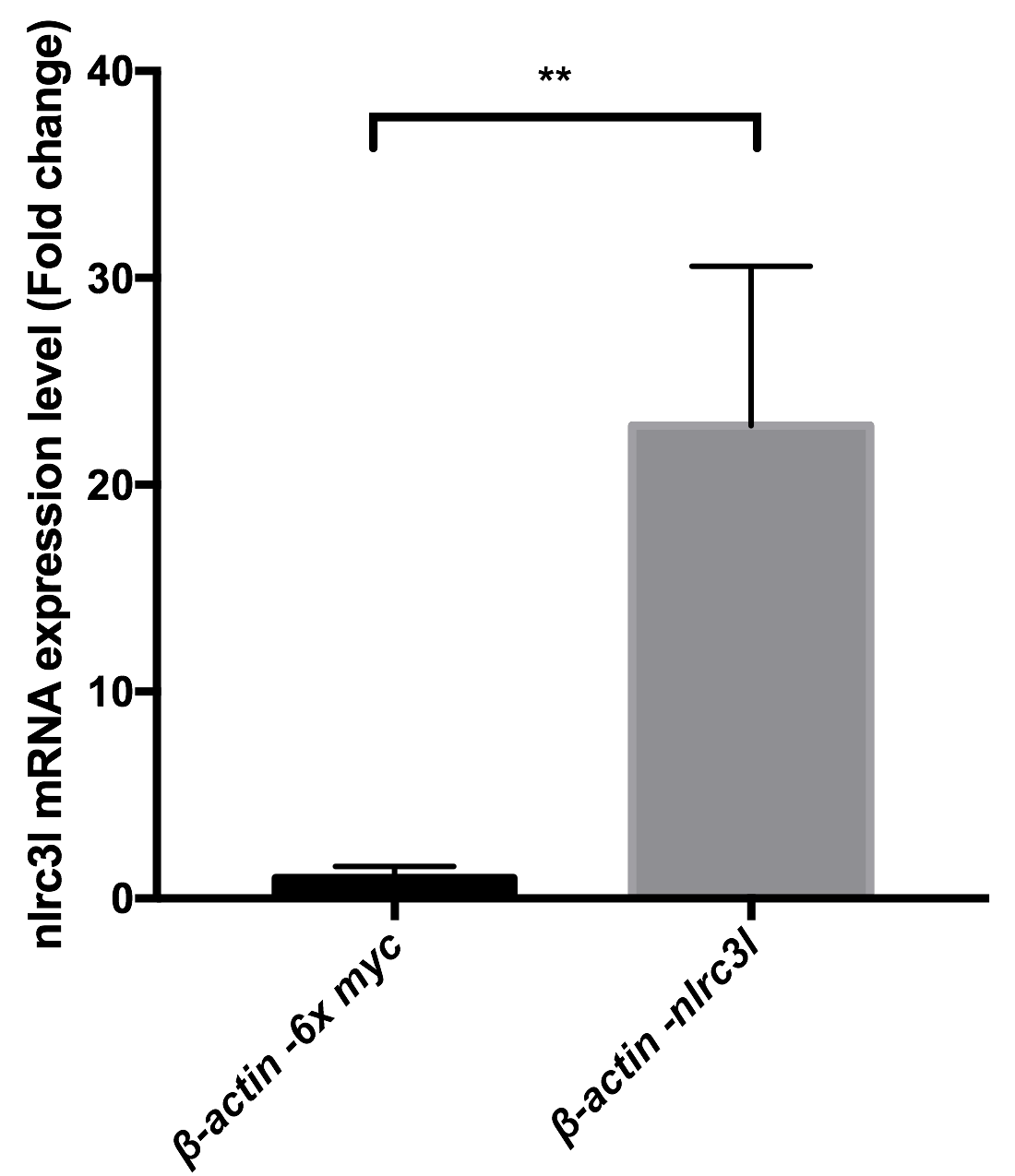
**

**Supplementary Figure 1. The *nlrc3-like* mRNA expression level in zebrafish embryos injected with transient expression plasmids, related to Figure 3.**

A: Quantitative RT-PCR showing expression of *nlrc3-like* in 3 dpf zebrafish embryos injected with *pTol2-β-actin-6xmyc (β-actin-6xmyc)* or *pTol2-β-actin-nlrc3-like* (*β-actin-nlrc3l*) at single cell stage. B: Quantitative RT-PCR showing expression of *nlrc3-like* in 3 dpf zebrafish embryos injected with *pTol2-coronin1a-6xmyc* (*coronin1a-6xmyc*) or *pTol2-coronin1a-nlrc3-like* (*coronin1a-nlrc3l*) at single cell stage. The data shown represents the averages from two independent biological replicates.

**Supplementary Figure 2. The expression patterns of inflammatory genes in macrophage after H37Rv infection, related to Figure 6.**

After Raw264.7 cells were infected by H37Rv (MOI=10), the expression of inflammatory genes mRNAs was measured at 0 hpi, 10 hpi, 24 hpi and 72 hpi.

**Supplementary Video 1. The recruitment of neutrophil to *M. marinum* in 3 dpf *nlrc3-like -/-* and non-mutant siblings zebrafish embryos, related to Figure 5.**

The video shows the migration behaviors of neutrophil (green) in 3 dpf *nlrc3-like -/-* and non-mutant siblings *Tg (lyz-GFP)* zebrafish embryos after the infect of *M. marinum:Tdtomato* (red) subcutaneously. The Z-stack time-lapse imagings were taken with a time interval of 1 min 30 s. Scale bar = 50 μm.
